# Gender Differences in Healthy Ranges for Serum Alanine Aminotransferase Levels in Adolescence

**DOI:** 10.1371/journal.pone.0021178

**Published:** 2011-06-27

**Authors:** Hossein Poustchi, Jacob George, Saeed Esmaili, Farzaneh Esna-Ashari, Gelayol Ardalan, Sadaf Ghajarieh Sepanlou, Seyed Moayed Alavian

**Affiliations:** 1 Digestive Disease Research Center, Tehran University of Medical Science, Tehran, Iran; 2 Storr Liver Unit, Westmead Millennium Institute, University of Sydney and Westmead Hospital, Sydney, Australia; 3 Department of Community and Preventive Medicine, Shaheed Beheshti University of Medical Sciences, Tehran, Iran; 4 Youth and School Health Office, Ministry of Health and Medical Education, Tehran, Iran; 5 Baqiyatallah Research Center for Gastroenterology and Liver Diseases (BRCGL), Baqiyatallah University of Medical Sciences, Tehran Hepatitis Center, Tehran, Iran; Aga Khan University, Pakistan

## Abstract

**Background & Aims:**

There is a worldwide epidemic of obesity among adolescents who subsequently are at increased risk for the development of non alcoholic fatty liver disease (NAFLD). The serum alanine aminotransferase (ALT) is the most frequently used test for screening these individuals, but no age and gender-specific upper limits of normal (ULN) based on healthy population data in children are available. The objective of the present study was to define ULN for ALT in healthy children in order to use this as a tool for case finding.

**Methods:**

A total of 975 school children (aged 7–18 years) were included in the study cohort. Highly significant correlations (all p<0.001) were noted between ALT values and measures of BMI, systolic and diastolic blood pressure, insulin levels, HOMA-IR, total cholesterol and triglyceride concentrations. In order to define the population with no risk factors, we excluded subjects having abnormal values for factors that correlated with ALT. This population comprised 186 boys and 185 girls.

**Results:**

In boys, median serum ALT levels were 16 IU/L and 9, 11, 18, and 30 IU/L for the 5th, 25th, 75th, and 95th percentiles. In girls, median serum ALT was 13, and 7, 9, 16, and 21 IU/L for the 5th, 25th, 75th, and 95th percentiles, respectively. The ULNs for ALT were 30 IU/L and 21 IU/L for boys and girls respectively. We found a linear relationship between age and ALT in females (p<0.001) but not in males. By multiple logistic regression, independent predictors of an elevated ALT included the BMI, waist hip ratio and levels of serum total cholesterol. In females, age was an additional inverse predictor.

**Conclusions:**

In children and adolescents, these normal limits for ALT should be applied. Those with persistent elevations should be investigated further.

## Introduction

There is a worldwide epidemic of obesity and diabetes, with an estimated 1 billion overweight adults [1]. Likewise, childhood obesity is increasing, with the development of end-organ damage in children and young adults [2], [3]. The spectrum of disorders related to adiposity is myriad, but includes insulin resistance, type 2 diabetes mellitus (T2DM), cardiovascular disease and fatty liver disease [4]–[9]. A central issue related to the management of obesity-related chronic diseases concerns laboratory screening and diagnosis. The serum alanine aminotransferase (ALT) activity correlates with obesity [10], reflects hepatocellular injury and is the most frequent test for screening and monitoring patients with non alcoholic fatty liver disease (NAFLD) [11]. Since NAFLD is a component of the metabolic syndrome and its consequences, it is not surprising that elevations in ALT activity are frequently present in persons with T2DM and cardiovascular disease and are associated with increased mortality [11]–[17].

Liver functions tests are the most frequently ordered tests in clinical practice, being relatively cheap and easy to measure. Of the panel, the ALT is the most specific screening test for hepatic necro-inflammation. Elevations in ALT activity usually reflect the presence of NAFLD, if other causes have been excluded. Data from pediatric studies indicate that NAFLD is an emergent problem, which in a proportion of cases, may progress to cirrhosis and liver-related morbidity and mortality [18]. Even in patients that do not develop advanced liver disease, an elevated ALT is an important surrogate for cardio-metabolic risk including dyslipidemia, hypertension and glucose intolerance. These patients are more likely to die of cardiovascular disease than from liver disease [19]–[25].

In screening for NAFLD, the quoted laboratory reference intervals for ALT serve as an important decision making tool [26], [27]. It has also been demonstrated that age and gender have significant effects on the levels of serum ALT and AST, with an inverted U pattern with respect to age [11], [28]–[30]. However, age and gender-specific upper limits of normal for the AST and ALT based on healthy population data in children are currently not available. Hence, the objective of the present study was to define upper limits of normal (ULN) for aminotransferases in healthy school age children in order to (1) define cut-offs for screening in primary and specialist health care settings and (2) to use this as a tool for early intervention in the management of fatty liver disease and as well, other complications of the metabolic syndrome.

## Results

Of 1000 students who consented to participate, 74 were excluded as they were unable to fast overnight. Another student was excluded after testing positive for hepatitis B surface antigen (HBsAg). None of students were positive for hepatitis C antibody and all subjects denied cigarette smoking or alcohol consumption. Hence, the data presented is based on a cohort of 925 individuals. Table 1 presents baseline demographic data for the whole cohort including age, gender BMI, HOMA-IR, blood pressure, ALT and AST level, and fasting glucose, fasting lipid profile and fasting insulin. The age and gender distribution of the students who agreed to participate in the study were no different from those that declined.

**Table 1:** Baseline characteristics of the total cohort and the ‘healthy’ population.

Variables	Whole cohort (n = 925)			Healthy subjects (371)		
	All mean (SD)	Male mean (SD)	Female mean (SD)	All mean (SD)	Male mean (SD)	Female mean (SD)
Age (yrs)	12.92 (3)	12.80 (2)	13 (3)	12.87 (3.13)	12.58 (3)	13.17 (3.41)
BMI (kg/m^2^)	20.45 (4.75)	19.89 (4.72)	20.88 (4.7)	18 (2.63)	17.59 (2.47)	18.41 (2.74)
HOMA-IR	3.12 (1.8)	3.15 (1.9)	3.08 (1.7)	2.43 (0.92)	2.43 (1.01)	2.43 (0.82)
SBP	10.53 (1.05)	10.8 (1.05)	10.33 (10)	10.21 (0.90)	10.39 (0.86)	10.03 (0.91)
DBP	6.50 (0.61)	6.65 (0.66)	6.38 (0.54)	6.35 (0.54)	6.47 (0.60)	6.23 (0.54)
ALT (IU/L)	17.21 (10.23)	19.96 (11.40)	15.01 (8.6)	14.84 (5.33)	16.63 (5.76)	13.04 (4.15)
AST (IU/L)	24.68 (12.47)	27.23 (8.44)	22.64 (14.62)	24.37 (6.39)	26.69 (6.43)	22.04 (5.45)
Fasting glucose (mg/dl)	81.05 (10.78)	82.95 (7.58)	79.53 (12.57)	80.13 (7.45)	81.99 (7.05)	78.25 (7.39)
Insulin(µU/ml)	15.30 (7.23)	14 (7.85)	15.54 (6.7)	12.22 (4.30)	11.90 (4.5)	12.54 (3.99)
T-C (mg/dl)	154.90 (27)	152.28 (26.9)	156.98 (26.97)	141.09 (16.95)	137.81 (16.21)	144.40 (17.08)
TG (mg/dl)	87.68 (41.51)	85.85 (42.18)	89.15 (40.95)	65.76 (15.73)	65.13 (16.29)	66.40 (15.16)
LDL (mg/dl)	88.55 (19.62)	86.32 (19.36)	90.32 (19.66)	79.97 (13.09)	77.50 (12.31)	82.45 (13.42)

Abbreviations: ALT: alanine aminotransferase, AST: aspartate aminotransferase, BMI: body mass index, HOMA-IR: homeostasis model assessment of insulin resistance, LDL: low-density lipoprotein, SD: standard deviation, T-C: Total cholesterol, TG: Triglyceride.

We next determined whether any of the clinical and laboratory parameters measured correlated with the values for serum ALT. As noted (Table 2), highly significant and positive correlations (all p<0.001) were noted between the serum ALT and measures of BMI, systolic and diastolic blood pressure, insulin levels, calculated HOMA-IR, total cholesterol and triglyceride concentrations. In contrast, there was a weak but inverse correlation between ALT and age (spearman r −0.077; p<0.021).

**Table 2:** Factors correlated with ALT level in 925 students.

	Age	BMI	SBP	DBP	Insulin	HOMA-IR	T-C	TG
Spearman	−0.077	0.169	0.198	0.181	0.136	0.124	0.148	0.164
P value	0.021	<0.001	<0.001	<0.001	<0.001	<0.001	<0.001	<0.001

Abbreviations: BMI: Body mass index; HOMA-IR: homeostasis model assessment of insulin resistance; DBP: diastolic blood pressure; SBP: systolic blood pressure; T-C: Total cholesterol; TG: Triglyceride.

In order to define the population with no risk factors for metabolic liver disease, we first excluded all subjects having abnormal values for factors correlated with ALT based on the definitions described earlier. This group, the *healthy population*, comprised of 186 boys and 185 girls and had values for fasting glucose, insulin levels, the total cholesterol and triglyceride within the normal range (80±7 mg/dl, 12±4 µU/ml, 141±17 mg/dl, 66±16 mg/dl, respectively. Table 1 lists the baseline characteristics of the total study cohort and of the healthy population.

We next defined the normal range and mean values for ALT and AST in these 371 normal subjects. Table 3 lists the median, mean and range for serum ALT and AST according to gender, and as well the percentiles for these values. In boys, serum ALT levels were 16 IU/L for the median, and 9, 11, 18, and 30 IU/L for the 5^th^, 25^th^, 75^th^, and 95^th^ percentiles, respectively. Serum AST levels were 26 for the median, and 17, 23, 30, and 38 IU/L for the 5^th^, 25^th^, 75^th^, and 95^th^ percentiles, respectively. In girls, serum ALT levels were 13 for the median, and 7, 8.6, 16, and 20.8 IU/L for the 5^th^, 25^th^, 75^th^, and 95^th^ percentiles, respectively. Serum AST levels were 21 for the median, and 14, 18, 25, and 32.7 IU/L for the 5^th^, 25^th^, 75^th^, and 95^th^ percentiles, respectively.

**Table 3:** Normal ranges for liver chemistry by gender in 371 healthy subjects.

	ALT (IU/L)		AST (IU/L)	
	Male (186)	Female (185)	Male (186)	Female (185)
Mean	16.63	13.05	26.7	22.05
Median	16	13	26	21
Min	8	5	4	13
Max	39	31	47	39
Percentile				
5	9	7	17	14
25	11	8.6	23	18
75	18	16	30	25
95	30	20.7	38	32.7

P<0.001 for difference in mean values of ALT and AST in males and females.

**Figure 1:**
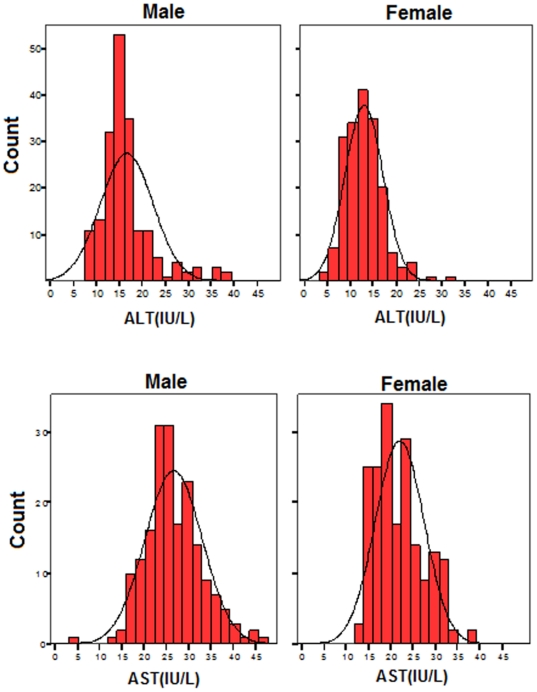
Histograms defining normal values for ALT and AST by gender in 371 healthy students.

Values below the gender-specific 95^th^ percentile were used to define the upper limit of normal value for the ALT and AST in boys and girls. Based on this definition, the ULNs for ALT were 30 IU/L and 21 IU/L for boys and girls respectively, while the ULNs for AST were 38 IU/L and 33 IU/L for boys and girls respectively. Gender-specific histograms of each of these values are presented in Figure 1.

**Figure 2:**
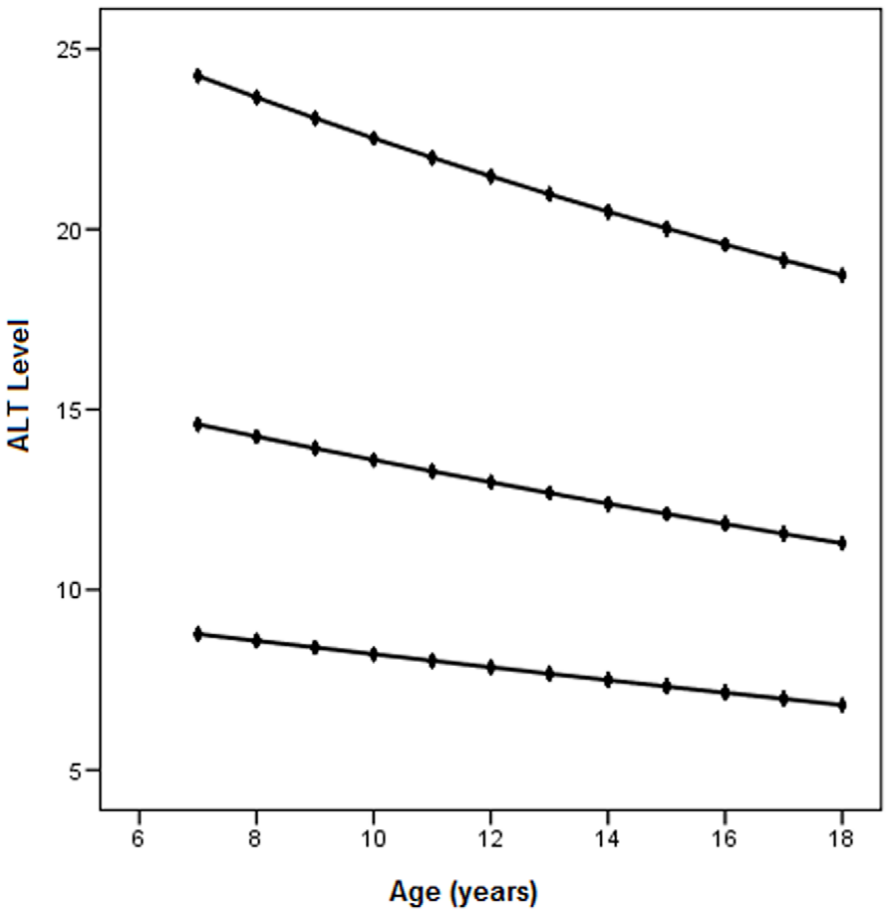
95^th^ percentile, predicted and 5th percentile of ALT values by age for females. The X axis represents current student age. The Y axis is the ALT level, and the top and bottom lines are the 95^th^ and 5^th^ percentile. The middle line represents the predicted values for ALT in female participants according to age.

In further analysis we found a linear relationship between age and ALT in females (p<0.001) but relationship for males was not significant (p = 0.41). Therefore, based on this finding we calculated 5th and 95th percentiles and predicted values for ALT by age in females (Figure 2).

We next categorized females into three age groups: group a) 7–10 years (n = 41), group b) 11–14 (n = 57) and group c) 15–18 years (n = 76) and calculated a separate range of ALT for each group. The minimum and maximum values for these three groups were 8, 8, 7 and 24, 22, 20 respectively for girls aged 7–10 years, 11–14 and 15–18 years.

As shown in Table 2, ALT values were correlated with several anthropometric and laboratory variables. In order to determine which factors were independently associated with elevations in ALT (segregated by gender), we undertook multiple logistic regression analysis with input variables that included age, BMI, SBP, DBP, Total TG, total cholesterol, HOMA-IR and LDL cholesterol. As shown in Table 4, independent predictors of an elevated ALT as expected included the BMI, the waist hip ratio and levels of serum total cholesterol. In females, age was an additional inverse predictor. Again, as shown in the table, visceral obesity as measured by the waist hip ratio was a stronger predictor of elevations in ALT (OR 2.25 CI1.4–3.56 in boys and 1.8 CI 1.16–2.8 in girls) than the BMI.

**Table 4:** Multiple logistic regressions for predictors of ALT by gender.

	Male			Female		
	OR	95% CI	P value	OR	95% CI	P value
Age (for each 10 yrs)	-	-	-	0.75	0.66–0.88	<0.001
Waist/hip ratio	2.25	1.4–3.56	<0.001	1.8	1.16–2.8	<0.001
BMI	2	1.68–2.37	0.001	1.61	1.34–1.94	<0.001
T-C	1.42	1.14–1.77	<0.001	1.32	1.09–1.62	<0.001

In the final analysis, we compared the number of components of the metabolic syndrome in the group with normal and elevated ALT levels based on ULNs calculated in the current study. As shown in Table 5, ALT values were similar between those who had only one component of the metabolic syndrome and those who did not (p = 0.385). In contrast, those with 2 or more components of the metabolic syndrome had elevated ALT levels (p<0.001). The metabolic syndrome was present in 7% of those with a normal ALT, but in 21% of those with an elevated ALT.

**Table 5:** Comparison of the frequency of the metabolic syndrome and its components in subjects with normal and elevated ALT based on ULNs calculated in the current study.

	Normal ALT (n = 711)	Elevated ALT(n = 99)	P value
1or more MetS components*	538(75.7%)	79 (79.8%)	0.358
2 or more MetS components	200 (28.1%)	45(45.5%)	<0.001
MetS	47 (6.67)	21 (21.2%)	<0.001

*MetS =  Metabolic Syndrome defined according to ATP III criteria [47].

## Discussion

In this study we undertook an age- and gender-based approach to define upper limits of normal for ALT and AST in school aged children and adolescents. In boys, ULNs for ALT and AST were 30 and 38 IU/L respectively. In girls the ULN for ALT and AST were 21 and 33 IU/L, respectively, and in this group ULNs for ALT (but not for AST) was inversely associated with age. In girls therefore, the ULN for ALT was 24 IU/L (age 7–10 years), 22 IU/L (11–14 years), and 20 IU/L (15–18 years). In multiple logistic regression analysis, serum total cholesterol, BMI and waist to hip ratio were independent predictors of an elevated ALT.

In defining reference intervals, identifying a group of healthy individuals is of paramount importance. For determining ULNs for ALT and AST therefore, subjects should be free of risk factors for liver diseases. In affluent nations, the most common liver disease in children and adolescents is NAFLD with a rate between 2.6% to 9.8% [31], [32]. The prevalence of NAFLD in the aforementioned age group in Iran is comparable, with a rate between 2.3% to 7.1% [33], [34]. Since Iran is located in an intermediate zone for HBV, we checked for hepatitis B surface antigen and to exclude HCV infection, anti-HCV antibody was assessed.

To determine ranges of ALT and AST in healthy children and adolescents, we next assessed for factors associated with serum ALT in univariate analysis. BMI, systolic and diastolic blood pressure, insulin levels, calculated HOMA-IR, the total cholesterol and triglyceride concentrations correlated with the serum ALT. Therefore, participants who had abnormal results for these components based on age-specific published values were excluded. In addition, those with elevated blood pressure or blood glucose were excluded and as discussed earlier, none of the subjects consumed alcohol or smoked cigarettes. Thus, the ULN values quoted for healthy children and adolescents in this study are likely to reflect true normative values at this age and are in accordance with studies published from elsewhere [35], [36]. Our sample size, we believe, is adequate for this purpose based on a consensus document produced by the Clinical and Laboratory Standards Institute (CLSI), formally known as the NCCLS [37]. They suggest that 120 is the minimum number of samples needed to establish a reference range to have a 90% confidence interval for the 97.5th percentile. Moreover if 198 samples are used then the confidence interval for the 97.5th percentile becomes 99%.

The upper limit of normal for ALT and AST in boys was higher than in girls. The reason for this is not known, but could relate to differences in muscle mass and sex hormones between genders. In this regard, it is well established that serum concentrations of estradiol are low in preadolescent girls and increase at menarche, while after menopause, serum concentrations decline to the levels below or similar to that in men [38]. These age-related hormonal changes could in part account for the reductions in the ULN of ALT that we observed in girls.

Considering the association between abnormal ALT values, cardiovascular disease, type 2 diabetes and NAFLD, our gender-specific reference ranges could help preventive health care systems in case ascertainment of children at higher risk of future morbidity. Consistent with this, we have shown that ALT levels were elevated in those with two or more components of the metabolic syndrome. Therefore, encountering elevated ALT values should prompt a thorough assessment of cardio-metabolic risk and the institution of preventive measures including lifestyle change and pharmacotherapy [32].

Our study has limitations in that we stratified normative ALT values in girls according to age, but had less than the 120 persons in each category as recommended by the Expert Panel on reference values [39]. However, studies such as the present one are difficult to undertake in children. Finally, segregation according to pubertal stages would have been ideal, but this was not ethically feasible.

In conclusion, we have used rigorous criteria to define normal values for serum aminotransferases in children. These limits should be routinely used when assessing children and adolescents. Further, we suggest that those with persistent elevations should be assessed for the metabolic syndrome and its components (if viral hepatitis has been excluded), and managed aggressively to improve long term health outcomes and quality of life.

## Methods

The study protocol was approved by the Ethics Council of the Iran Ministry of Health and Medical Education. The study population was selected using a stratified multistage random sampling design according to the age, gender and geographic location of children from the city of Tehran, Iran. For this study, Tehran was divided into 5 geographic regions. Written invitations were sent to 11,000 randomly selected students from public school registries in the 5 regions. A stratified cluster random sampling design was conducted on those students who responded. Ultimately, 1,000 students aged 7–18 were enrolled in the study. Written informed consent was obtained from parents and oral consent from students after full explanation of the procedures. For blood sampling, students were invited to the Children's Medical Centre and one of the parents accompanied his/her child. Each student was initially assessed by a trained nurse who administered a questionnaire to obtain information on demographic variables, the medical history, dietary habits, alcohol consumption and cigarette smoking. Ten ml of venous blood was taken from an ante-cubital vein in clotted tubes, placed in ice and transported to the reference laboratory. All laboratory assessments were performed on the same day. All subjects subsequently underwent a liver ultrasound by an expert radiologist.

Body weight was measured in all students in the upright position to the nearest 0.1 kg while wearing light clothing without shoes. Height was measured to the nearest millimeter with a portable stadiometer. Waist circumference was measured at the end of normal expiration to the nearest millimeter, at the narrowest point between the lower borders of the rib cage and the iliac crest. Hip circumference was obtained at the widest point between hip and buttock.

Blood pressure was measured using a mercury sphygmomanometer after confirming that subjects did not consume caffeine or smoke cigarettes 30 min prior to the measurement. All subjects were seated for at least 5 minutes. The first and fifth Korotkoff sounds were recorded as the systolic blood pressure (SBP) and the diastolic blood pressure (DBP), respectively. This was measured twice and the mean used in the analysis.

Plasma concentrations of glucose, total cholesterol, triglyceride, high density lipoprotein (HDL) cholesterol, AST, ALT and insulin were measured using an auto analyzer (Hitachi 912 auto-analyzer, Tokyo, Japan). All subjects were checked for hepatitis B surface antigen (HBsAg) and anti-HCV antibody (HCVAb).

The body mass index (BMI) was calculated as weight divided by the height in meters squared (kg/m^2^). Gender-specific BMI cut-off values based on 6 nationally representative data sets were used to define overweight and obesity in children [40].

Normotensive systolic and diastolic blood pressure was defined as the National High Blood Pressure Education Program (NHBPEP) recommended cut-point of the 90^th^ percentile for age, gender, and height [41]. A triglyceride (TG) concentration ≥100 mg/dl was considered elevated based on the criteria used to define the metabolic syndrome in adolescents [42]. High density lipoprotein-cholesterol (HDL-C) <50 mg/dl was used to define a low HDL cholesterol, except in boys aged 15–18 years, in whom <45 mg/dl was used as the definition for low HDL cholesterol [42]. Fasting glucose ≥100 mg/dl was classified as elevated based on the recommendations of the American Diabetes Association (2003) and the WHO (2006) [43], [44]. The cut-offs for total cholesterol (T-C <170 mg/dl) and low density lipoprotein-cholesterol (LDL-C <110 mg/dl) were accepted as normal [45]. Insulin resistance was calculated using the homeostasis model assessment of insulin resistance (HOMA-IR) [46]and insulin resistance was defined as HOMA-IR >2 [23]. We used NCEP-ATP III criteria to define the metabolic syndrome [47].

Fatty liver was diagnosed on an abdominal ultrasound performed in all subjects using an ALOKA SSD 1700 machine by an experienced radiologist, unaware of the laboratory and other results. The presence of fatty liver was determined using accepted criteria that included a diffuse increase in echo texture (bright liver), increased liver echo texture compared with the kidneys, vascular blurring and deep attenuation [48].

All data were analyzed using SPSS for Windows Version 15 (SPSS Inc, Chicago, USA). The Student's *t* test and one-way ANOVA were used for continuous variables. Non-parametric tests were used when parametric assumptions were not met. Independent predictors of ALT were identified by multiple logistic regression analysis, with backward stepwise variable selection. A p-value of ≤0.05 denoted significance.

The Medical Ethics Committee in Digestive Disease Research Center approved the conduct of the current study. Study was conducted in accord with principles of Declaration of Helsinki. Informed consent was signed by guardians of all participating adolescents. Data were de-identified and the personnel were blind to the identity of subjects.
